# Duration of total contact casting for resolution of acute Charcot foot: a retrospective cohort study

**DOI:** 10.1186/s13047-021-00477-5

**Published:** 2021-06-15

**Authors:** Danielle A. Griffiths, Michelle R. Kaminski

**Affiliations:** 1 Department of Podiatry, Eastern Health, Melbourne, Victoria 3135 Australia; 2 Discipline of Podiatry, School of Allied Health, Human Services and Sport, La Trobe University, Melbourne, Victoria 3086 Australia; 3 Department of Podiatry, St Vincent’s Hospital Melbourne, Melbourne, Victoria 3065 Australia

**Keywords:** Charcot foot, Diabetic foot, Foot deformities, Neurogenic arthropathy, Retrospective studies

## Abstract

**Background:**

Charcot neuroarthropathy (Charcot foot) is a highly destructive joint disease of the foot and ankle. If there is delayed diagnosis and treatment, it can lead to gross deformity, instability, recurrent ulceration and/or amputation. Total contact casting (TCC) is a treatment commonly used to immobilise the foot and ankle to prevent trauma, further destruction and preserve the foot structure during the inflammatory phase. At present, there is limited Australian data regarding the duration of TCC treatment for resolution of acute Charcot foot, and whether there are any patient and clinical factors affecting its duration. Therefore, this study aimed to address these deficiencies.

**Methods:**

This study presents a retrospective analysis of 27 patients with acute Charcot foot attending for TCC treatment at a high-risk foot service (HRFS) in a large metropolitan health network in Melbourne, Australia. Over a three-year period, data were retrospectively collected by reviewing hospital medical records for clinical, demographic, medical imaging and foot examination information. To explore between-group differences, independent samples *t*-tests, Mann-Whitney *U* tests, Chi-square tests, and/or Fisher’s exact tests were calculated depending on data type. To evaluate associations between recorded variables and duration of TCC treatment, mean differences, odds ratios (OR) and 95% confidence intervals were calculated.

**Results:**

Mean age was 57.9 (SD, 12.6) years, 66.7% were male, 88.9% had diabetes, 96.3% had peripheral neuropathy, and 33.3% had peripheral arterial disease. Charcot misdiagnosis occurred in 63.0% of participants, and signs and symptoms consistent with acute Charcot foot were present for a median of 2.0 (IQR, 1.0 to 6.0) months prior to presenting or being referred to the HRFS. All participants had stage 1 Charcot foot. Of these, the majority were located in the tarsometatarsal joints (44.4%) or midfoot (40.7%) and were triggered by an ulcer or traumatic injury (85.2%). The median TCC duration for resolution of acute Charcot foot was 4.3 (IQR, 2.7 to 7.8) months, with an overall complication rate of 5% per cast. Skin rubbing/irritation (40.7%) and asymmetry pain (22.2%) were the most common TCC complications. Osteoarthritis was significantly associated with a TCC duration of more than 4 months (OR, 6.00). Post TCC treatment, 48.1% returned to footwear with custom foot orthoses, 25.9% used a life-long Charcot Restraint Orthotic Walker, and 22.2% had soft tissue or bone reconstructive surgery. There were no Charcot recurrences, however, contralateral Charcot occurred in 3 (11.1%) participants.

**Conclusions:**

The median TCC duration for resolution of acute Charcot foot was 4 months, which is shorter or comparable to data reported in the United Kingdom, United States, Europe, and other Asia Pacific countries. Osteoarthritis was significantly associated with a longer TCC duration. The findings from this study may assist clinicians in providing patient education, managing expectations and improving adherence to TCC treatment for acute Charcot neuroarthropathy cases in Australia.

**Supplementary Information:**

The online version contains supplementary material available at 10.1186/s13047-021-00477-5.

## Background

Charcot neuroarthropathy (Charcot foot) is a highly destructive joint disease characterised by progressive multiple bone fractures, dislocations and severe deformity of the foot and ankle [1–3]. There are a number of medical conditions with neuropathic manifestations that are linked to the development of Charcot foot [4], although diabetes has become the leading cause in the Western world [3–6]. The estimated prevalence of Charcot foot in the general diabetes population is 0.08%, but can rise to 13% in high-risk foot populations [3, 4]. Acute Charcot neuroarthropathy typically presents as a warm, erythematous and oedematous foot [7, 8]. As such, this condition is often masked by, or is clinically indistinguishable from deep vein thrombosis, inflammatory arthritis (e.g. gout) or infection (e.g. cellulitis, osteomyelitis). Consequently, this often leads to delayed or missed diagnosis in its early stages [4, 7, 9].

Delayed diagnosis can result in devastating complications such as gross deformity, instability, recurrent ulceration and/or amputation [2, 3, 6, 9]. Structural damage to the foot and ankle can be limited, however, if acute Charcot foot is identified and managed early [2, 7, 9]. Total contact casting (TCC) is a treatment used to offload and immobilise the affected foot and ankle to prevent trauma, further fractures and destruction, and preserve the foot structure during the inflammatory phase [10, 11]. The TCC slows the progression of the Charcot foot by controlling the cycle of inflammation and the degree of lower limb oedema [3, 5, 8, 11, 12].

It is widely accepted that TCC is the preferred method of immobilisation for an acute Charcot foot, as it is customised and non-removable [3, 10, 12]. However, a common question from patients being treated with TCC is “*this cast is hot and heavy; how long do I have to use it?*” Duration of TCC treatment will depend upon the patient’s response, but it is recommended that casting is continued until the temperature differential is less than 2 °C between the affected and non-affected Charcot foot for 4–6 consecutive weeks, and there are radiographic signs of healing [3, 8, 13–16].

Globally, population-based studies have found significant discrepancies regarding the duration of TCC treatment for resolution of acute Charcot foot. In the United Kingdom (UK), studies have reported treatment times of 9 to 12 months [2, 17, 18], while data from the United States (US) [10, 11, 19, 20], Europe [21–28] and other Asia Pacific countries [29, 30] have reported shorter treatment times of 3 to 9 months. Unfortunately, none of the above-mentioned studies included Australian subjects. To date, there has only been one Australian study that has reported on TCC duration times for acute Charcot foot resolution. This study reported an average treatment time of approximately 10 months [31]. Therefore, there is limited data regarding the duration of TCC treatment for resolution of acute Charcot foot in the Australian population. To address this deficiency, this study conducted a retrospective analysis of cases presenting to a high-risk foot service (HRFS) with acute Charcot foot over a three-year period. This study specifically aimed to investigate the time to resolution of acute Charcot foot with TCC treatment, and to explore patient and clinical factors affecting its duration.

## Methods

### Ethics approval

The Human Research and Ethics Committee of Eastern Health (reference number: LR25/2015) approved the study. Informed consent was not required of participants due to the retrospective nature of the study, and data collection involved the use of existing medical records only.

### Study design

This retrospective cohort study was conducted in a HRFS at a large metropolitan health network in Melbourne, Australia from 4 January 2012 to 4 January 2015. Clinical, demographic, medical imaging, and foot examination information were retrospectively collected from existing medical records and from medical imaging/pathology reports (Additional file 1).

### Participants

All adults attending the HRFS over the three-year period with an acute Charcot foot (defined as modified Eichenholtz stage 0 or 1 [16, 32, 33]) were eligible for inclusion. Participants were excluded if they had a chronic Charcot foot (defined as modified Eichenholtz stage 2 or 3 [16, 32, 33]), concurrent rheumatic arthropathy, rheumatoid arthritis, psoriatic arthritis, gout, systemic lupus erythematosus, erysipelas, cellulitis, and/or osteomyelitis, had a TCC for management of a foot ulcer or fracture (i.e. not related to Charcot), or if they transferred/withdrew from the HRFS prior to resolution of their acute Charcot foot.

### Data collection

Initially, all records from the HRFS were screened for eligibility based on the inclusion and exclusion criteria. One investigator (D.G.) reviewed hospital medical records for information relating to participant characteristics, comorbidities, foot assessment data, Charcot foot history, and TCC treatment. Data were checked for accuracy by another investigator (M.R.K.). The screening tool and data collection form used in this study can be found in Additional file 1.

Peripheral neuropathy was defined as a Semmes-Weinstein 5.07/10 g monofilament score < 3/3 over the plantar aspects of the hallux, 3rd and 5th metatarsals on either foot [34]. Peripheral arterial disease was defined as absence of ≥2 pedal pulses [35], toe-brachial pressure index ≤0.6, and/or ankle-brachial pressure index ≤0.9 either foot [35, 36].

Acute Charcot foot (defined as modified Eichenholtz stage 0 or 1 [16, 32, 33]) was documented when at least one or more of the following clinical features were evident in the medical records AND there were conclusive diagnostic imaging findings:

- Clinical signs/symptoms (i.e. erythema, oedema, increased temperature, bounding pulses) ± history of trauma or surgery ± history of pain ± structural deformity [3] or;
- More than 2 °C dermal temperature differential between the suspected Charcot foot and the contralateral foot [2, 3, 5, 13–15, 22, 37] or;
- Peripheral neuropathy (i.e. score of < 3/3 on either foot with a 10 g monofilament test) [1, 3, 5, 34, 38] and;
- Conclusive diagnostic imaging findings (e.g. osteopenia, fragmentation, joint subluxation, fractures) [16].

Charcot foot pattern was recorded according to its anatomical site(s) of involvement using the Sanders and Frykberg classification [6, 39]. Charcot foot history including duration of signs and symptoms consistent with acute Charcot foot prior to attending the HRFS, Charcot misdiagnosis, and potential Charcot triggers were recorded. This information was obtained via patient report within the HRFS progress notes.

**Fig. 1:**
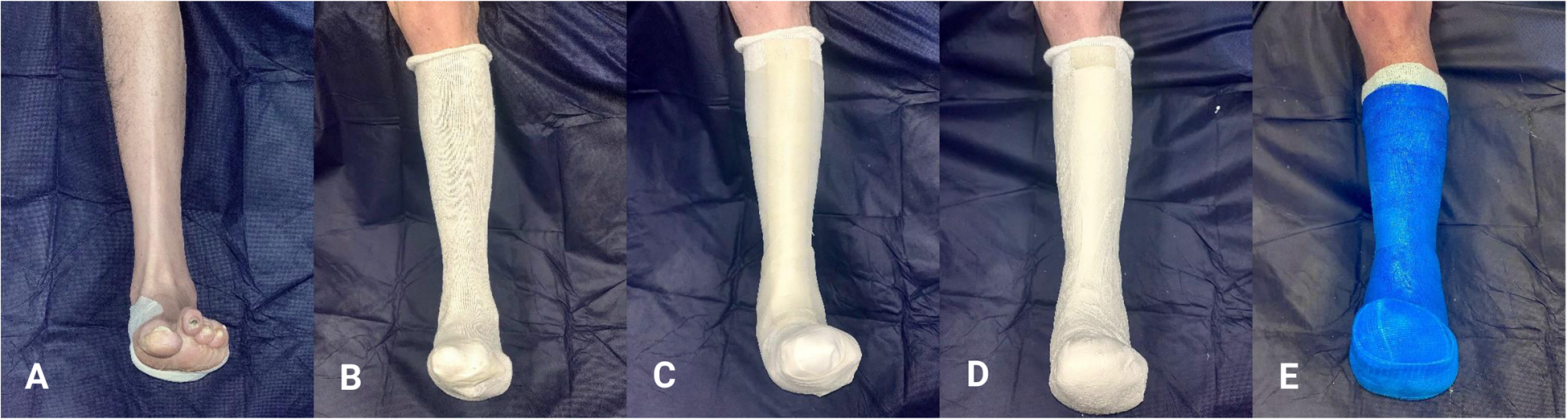
**Application of total contact cast.** (**A**) Felt padding (‘D-filler’) is placed under medial longitudinal arch (except in cases of severe ‘rockerbottom’ deformity). (**B**) Cotton tube bandage is applied to protect lower limb and foot. (**C**) Silicone strips are applied over bony prominences. (**D**) Plaster of paris inner layer is applied. (**E**) Fibreglass outer layer is applied. Participant photographs printed with permission

The HRFS in this study used the following technique [40] for application of the serial TCCs (Fig. 1):

- 10–20 mm felt padding (i.e. D-filler) placed under the medial longitudinal arch (no felt padding applied in cases of severe ‘rockerbottom’ Charcot deformity);
- Cotton tube bandage applied to cover and protect the lower limb;
- 5 mm silicone strips cut to size and applied over bony prominences (e.g. anterior tibia, medial/lateral malleoli);
- Inner layer of plaster of paris applied, followed by an outer splint of fibreglass;
- Decision regarding toes in or out is made on an individual basis (e.g. personal preference, feelings of claustrophobia);
- Post-operative shoe (e.g. Darco®) used over TCC for protection during ambulation and;
- Patients may ambulate immediately post TCC application but are encouraged to avoid weightbearing on the affected side.

Duration of TCC treatment was defined as the number of days between the date of the first TCC application to the date of TCC cessation for the same episode of acute Charcot foot. In cases of unexpected removal of TCC (e.g. patient request, work/travel requirements), the time spent out of the TCC was included in the participant’s overall TCC treatment time.

The decision to cease TCC treatment in the HRFS is guided by clinical assessment performed by experienced clinicians in Orthopaedics and Podiatry. Changes noted during clinical assessment include visual signs of reduced oedema, erythema and skin temperatures. Objective and quantifiable temperature testing is conducted using a hand-held infrared dermal thermometer (Exergen Corporation® ‘DermaTemp-1001’). After a 15-min acclimatisation period (i.e. following removal of the TCC and contralateral footwear/hosiery) [41], ten anatomical sites (plantar 1st/3rd/5th metatarsal heads, plantar aspect of base of 5th metatarsal [styloid process], dorsal aspect of base of 3rd metatarsal, medial aspect of base of 1st metatarsal, medial aspect of navicular, plantar medial tubercle of calcaneus, medial malleolus and lateral malleolus) are tested weekly, then extended out to fortnightly when oedema has stabilised [42].

In the HRFS, resolution of acute Charcot foot is determined based on the following:

- Modified Eichenholtz stage 3 or 4 Charcot foot [16, 32, 33] confirmed by medical imaging and/or;
- Dermal temperature differential less than 2 °C for 6 consecutive weeks for all anatomical testing sites [3].

Resolution of acute Charcot foot is then followed by transition into a Charcot Restraint Orthotic Walker (CROW), knee-high removable offloading boot (e.g. Controlled Ankle Motion [CAM] walker) or therapeutic footwear with custom foot orthoses [40].

### Data handling and statistical analysis

Data were entered into a Microsoft Excel® spreadsheet for the development of an analytical file. Participant data were initially checked for duplication (i.e. for those with multiple admissions to the HRFS). To ensure confidentiality and privacy of participants, all data were made non-identifiable with the use of a coding system. Participant characteristics were calculated and expressed as mean (standard deviation, SD) or median (interquartile range, IQR, 25th to 75th percentile) for continuous variables and number (proportion) for categorical variables. Continuous data were checked for normality. To explore between-group differences (for example, TCC duration < 4 or > 4 months), independent samples *t*-tests, Mann-Whitney *U* tests, Chi-square tests, and/or Fisher’s exact tests were calculated depending on data type. To evaluate associations between recorded variables and duration of TCC treatment, mean differences, odds ratios (OR) and 95% confidence intervals (CI) were calculated. IBM SPSS version 26.0 (IBM Corp, Somers, NY, USA) was used for statistical analysis.

## Results

Of the 46 patient records screened, 27 adults with acute Charcot foot attending the HRFS between January 2012 and January 2015 were eligible for inclusion. Table 1 provides the participant characteristics. The mean age (SD) was 57.9 (12.6) years, 66.7% were male, and 29.6% had a smoking history (i.e. past or current smoker). The majority of participants had diabetes (88.9%) with an average duration of 24.0 (SD, 13.3) years and glycated haemoglobin of 8.6% (SD, 2.0). Overall, there was a high prevalence of previous foot complications including foot ulceration (66.7%), infection (63.0%) and amputation (14.8%). Peripheral neuropathy and peripheral arterial disease were present in 96.3 and 33.3%, respectively (Table 2).

**Table 1:** Participant characteristics

		Median TCC duration			
Variable	Total (N = 27)	< 4 months (***n*** = 12)	> 4 months (***n*** = 15)	MD (95% CI)^†^ or OR (95% CI)	***P***-value*
Mean age (SD), *years*	57.9 (12.6)	56.3 (13.7)	59.1 (12.0)	2.81 (−7.40 to 13.02)^†^	0.576
Male sex, *n (%)*	18 (66.7)	8 (66.7)	10 (66.7)	1.00 (0.20 to 5.00)	> 0.99
Known ethanol abuse, *n (%)*	6 (22.2)	4 (33.3)	2 (13.3)	0.31 (0.05 to 2.08)	0.357
Smoking history, *n (%)*^a^	8 (29.6)	2 (16.7)	6 (40.0)	3.33 (0.53 to 20.91)	0.236
Diabetes, *n (%)*	24 (88.9)	10 (83.3)	14 (93.3)	2.80 (0.22 to 35.29)	0.569
Type 1, *n (%)*	8 (33.3)	4 (40.0)	4 (28.6)	0.60 (0.11 to 3.34)	0.673
Type 2, *n (%)*	16 (66.7)	6 (60.0)	10 (71.4)	1.67 (0.30 to 9.27)	0.673
Mean duration (SD), *years*	24.0 (13.3)	22.4 (13.5)	25.1 (13.6)	2.74 (−8.87 to 14.36)^†^	0.629
Mean HbA1c (SD), *%*^b^	8.6 (2.0)	8.1 (2.5)	8.9 (1.7)	0.82 (−0.99 to 2.63)^†^	0.358
Dyslipidaemia, *n (%)*	16 (59.3)	6 (50.0)	10 (66.7)	2.00 (0.42 to 9.52)	0.452
Hypertension, *n (%)*	13 (48.1)	4 (33.3)	9 (60.0)	3.00 (0.62 to 14.62)	0.168
Ischaemic heart disease, *n (%)*	3 (11.1)	1 (8.3)	2 (13.3)	1.69 (0.14 to 21.27)	> 0.99
Congestive cardiac failure, *n (%)*	0 (0)	0 (0)	0 (0)	N/A	N/A
Cerebrovascular disease, *n (%)*	2 (7.4)	0 (0)	2 (13.3)	1.92 (1.32 to 2.80)	0.487
Chronic kidney disease, *n (%)*	4 (14.8)	1 (8.3)	3 (20.0)	2.75 (0.25 to 30.51)	0.605
Osteoarthritis, *n (%)*	13 (48.1)	3 (25.0)	10 (66.7)	6.00 (1.11 to 32.55)	0.031*
Previous foot ulceration, *n (%)*	18 (66.7)	7 (58.3)	11 (73.3)	1.96 (0.39 to 9.93)	0.448
Previous foot infection, *n (%)*	17 (63.0)	7 (58.3)	10 (66.7)	1.43 (0.30 to 6.88)	0.706
Previous amputation, *n (%)*	4 (14.8)	3 (25.0)	1 (6.7)	0.21 (0.02 to 2.39)	0.294

Data are n (%), MD (95% CI) or OR (95% CI), unless otherwise specified. Percentages may not add up to 100%, as they are rounded to the nearest percent

*CI*, confidence interval; *HbA1c*, glycated haemoglobin; *MD*, mean difference; *N/A*, not applicable; *OR*, odds ratio; *SD*, standard deviation; *TCC*, total contact casting

SI conversion factor: To convert HbA1c to proportion of total haemoglobin, multiply by 0.01

^*^Significant difference between ‘< 4 months’ and ‘> 4 months’ median TCC duration groups, *p* < 0.05

^a^Past or current smoker

^b^Maximum missing data were for glycated haemoglobin (HbA1c) involving 1 participant overall (3.7%)

^†^Data are MD (95% CI), unless otherwise specified

**Table 2:** Neurovascular foot assessments

		Median TCC duration			
**Foot assessment/variable**	**Total** **(*N*** **= 27)**	**< 4 months** **(*n*** **= 12)**	**> 4 months** **(*n*** **= 15)**	**OR (95% CI)**	*P*-value*
Peripheral neuropathy^a^	26 (96.3)	11 (91.7)	15 (100.0)	0.42 (0.27 to 0.66)	0.444
Left					
0/3, *n (%)*	24 (88.9)	11 (84.6)	13 (92.9)		
1/3, *n (%)*	1 (3.7)	0 (0)	1 (7.1)		
2/3, *n (%)*	1 (3.7)	1 (7.7)	0 (0)		
3/3, *n (%)*	1 (3.7)	1 (7.7)	0 (0)		
Right					
0/3, *n (%)*	24 (88.9)	11 (84.6)	13 (92.9)		
1/3, *n (%)*	1 (3.7)	1 (7.7)	0 (0)		
2/3, *n (%)*	0 (0)	0 (0)	0 (0)		
3/3, *n (%)*	2 (7.4)	1 (7.7)	1 (7.1)		
Peripheral arterial disease^b^	9 (33.3)	4 (33.3)	5 (33.3)	1.00 (0.20 to 5.00)	> 0.99
Pedal pulses^†^					
0/4, *n (%)*	1 (3.7)	1 (7.7)	0 (0)		
1/4, *n (%)*	1 (3.7)	0 (0)	1 (7.1)		
2/4, *n (%)*	4 (14.8)	2 (15.4)	2 (14.3)		
3/4, *n (%)*	1 (3.7)	0 (0)	1 (7.1)		
4/4, *n (%)*	19 (70.4)	10 (76.9)	9 (64.3)		
Mean ABPI (SD)					
Left^†^	1.15 (0.20)	1.11 (0.21)	1.20 (0.20)		
Right^†^	1.11 (0.23)	1.08 (0.15)	1.16 (0.33)		
Mean TBPI (SD)					
Left^†^	0.82 (0.15)	0.79^c^	0.83 (0.21)		
Right^†^	0.66 (0.17)	0.78^c^	0.53^c^		
Mean systolic toe pressure (SD)					
Left^†^	105.4 (11.6)	118^c^	102.3 (10.6)		
Right^†^	99.0 (13.9)	109^c^	95.7 (15.0)		

Data are n (%) or mean (SD), unless otherwise specified. Percentages may not add up to 100%, as they are rounded to the nearest percent

*ABPI*, ankle-brachial pressure index; *SD*, standard deviation; *TBPI*, toe-brachial pressure index; *TCC*, total contact casting

^*^Significant difference between ‘< 4 months’ and ‘> 4 months’ median TCC duration groups, p < 0.05

^a^Peripheral neuropathy was defined as a monofilament score < 3/3 either foot

^b^Peripheral arterial disease was defined as absence of ≥2 pedal pulses, TBPI ≤0.6, and/or ABPI ≤0.9 either foot

^†^Maximum missing data were for right TBPI involving 25 participants overall (92.6%). Missing data were for pedal pulses (n = 1), ABPI (n = 15), left TBPI (*n* = 24), right systolic toe pressure (*n* = 23), and left systolic toe pressure (*n* = 20)

^c^As a result of missing data pertaining to toe systolic pressures, ankle systolic pressures and/or TBPI scores, data reported are the raw data for one participant only. Therefore, mean (SD) were unable to be calculated and reported

Charcot misdiagnosis occurred in 63.0% of participants, and signs and symptoms consistent with acute Charcot foot were present for a median of 2.0 (IQR, 1.0 to 6.0) months prior to presenting or being referred to the HRFS. The affected foot was most commonly misdiagnosed as cellulitis (47.1%). All participants presented with a stage 1 Charcot foot. Of these, the majority were located in the tarsometatarsal joints (44.4%) or midfoot (40.7%) and were triggered by an ulcer or traumatic injury (85.2%). All participants received x-ray imaging to establish diagnosis at initial presentation to the HRFS, while 14.8% also had a bone scan and/or magnetic resonance imaging (Table 3).

**Table 3:** Charcot foot history

		Median TCC duration			
Variable	Total (N = 27)	< 4 months (n = 12)	> 4 months (n = 15)	OR (95% CI)	***P***-value*
Charcot foot					
Left, *n (%)*	14 (51.9)	4 (33.3)	10 (66.7)	4.00 (0.80 to 20.02)	0.085
Right, *n (%)*	13 (48.1)	8 (66.7)	5 (33.3)	0.25 (0.05 to 1.25)	0.085
Stage of Charcot					
Stage 0, *n (%)*	0 (0)	0 (0)	0 (0)	N/A	N/A
Stage 1, *n (%)*	27 (100.0)	12 (100.0)	15 (100.0)	N/A	N/A
Median Charcot duration (IQR), *months*^a,b^	2.0 (1.0 to 6.0)	2.5 (1.0 to 6.0)	1.8 (0.9 to 6.8)	N/A	0.709^†^
Charcot trigger					
Ulceration, *n (%)*	9 (33.3)	5 (41.7)	4 (26.7)	0.51 (0.10 to 2.57)	0.448
Injury/trauma, *n (%)*	14 (51.9)	5 (41.7)	9 (60.0)	2.10 (0.45 to 9.84)	0.343
Amputation, *n (%)*	2 (7.4)	2 (16.7)	0 (0)	1.20 (0.93 to 1.55)	0.188
Lymphoedema, *n (%)*	1 (3.7)	0 (0)	1 (6.7)	0.93 (0.82 to 1.07)	> 0.99
Unknown, *n (%)*	1 (3.7)	0 (0)	1 (6.7)	0.93 (0.82 to 1.07)	> 0.99
Charcot misdiagnosis, *n (%)*	17 (63.0)	7 (58.3)	10 (66.7)	1.43 (0.30 to 6.88)	0.706
Charcot pattern					
Tarsometatarsal joints, *n (%)*	12 (44.4)	5 (41.7)	7 (46.7)	1.23 (0.27 to 5.67)	0.795
NC, TN, CC joints, *n (%)*	11 (40.7)	6 (50.0)	5 (33.3)	0.50 (0.11 to 2.38)	0.452
Ankle and subtalar joints, *n (%)*	1 (3.7)	0 (0)	1 (6.7)	0.93 (0.82 to 1.07)	> 0.99
Combination, *n (%)*^c^	3 (11.1)	1 (8.3)	2 (13.3)	1.69 (0.14 to 21.3)	> 0.99
Imaging received					
X-ray, *n (%)*	27 (100.0)	12 (100.0)	15 (100.0)	N/A	N/A
Bone scan, *n (%)*	4 (14.8)	0 (0)	4 (26.7)	0.73 (0.54 to 1.00)	0.106
MRI, *n (%)*	4 (14.8)	2 (16.7)	2 (13.3)	0.77 (0.09 to 6.45)	> 0.99

Data are n (%) or OR (95% CI), unless otherwise specified. Percentages may not add up to 100%, as they are rounded to the nearest percent

*CC*, calcaneocuboid; *CI*, confidence interval; *IQR*, interquartile range; *MRI*, magnetic resonance imaging; *N/A*, not applicable; *NC*, naviculocuneiform; *OR*, odds ratio; *SD*, standard deviation; *TCC*, total contact casting; *TN*, talonavicular

^*^Significant difference between ‘< 4 months’ and ‘> 4 months’ median TCC duration groups, p < 0.05

^a^Approximate duration of signs and symptoms consistent with acute Charcot foot prior to attending the high-risk foot service. This information was obtained via patient report within the clinic

^b^Maximum missing data were for Charcot duration involving 3 participants overall (11.1%)

^c^Two participants (7.4%) had a combination Charcot pattern involving the tarsometatarsal joints and the NC, TN and CC joints. One participant (3.7) had a combination Charcot pattern involving the calcaneus and the forefoot

^†^*P*-value relates to Mann-Whitney *U* test

Throughout the study period, there were a total of 421 TCCs applied (average of 15 TCCs per participant). The median TCC duration for resolution of acute Charcot foot was 4.3 (IQR, 2.7 to 7.8) months. A large proportion of participants (77.8%) were able to weightbear as tolerated using a post-operative shoe over the TCC. Overall, there were 21 complications/adverse events related to the TCC treatment, the majority being minor and reversible. Skin rubbing/irritation (40.7%) and asymmetry pain (22.2%) were the most common. The overall complication rate was 5% per cast (calculated from the total number of complications divided by the total number of TCCs i.e. 21/421). Post TCC treatment, almost half of the participants (48.1%) transitioned into specialised or off-the-shelf footwear with custom foot orthoses, 25.9% used a life-long CROW, and 22.2% underwent soft tissue or bone reconstructive surgery. The median follow-up time from ceasing TCC treatment (due to Charcot resolution) to the end of the study period was 11.9 (IQR, 2.8 to 14.6) months. During this time, there were no recurrent Charcot feet recorded, however, contralateral Charcot foot occurred in 3 (11.1%) participants (Table 4).

**Table 4:** Total contact casting treatment

		Median TCC duration			
Variable	Total (N = 27)	< 4 months (***n*** = 12)	> 4 months (***n*** = 15)	OR (95% CI)	***P***-value*
TCC applications					
Total number	421	101	320	N/A	N/A
Mean (SD), *per participant*	15.6 (9.2)	8.4 (2.3)	21.3 (8.5)	N/A	N/A
TCC duration					
Median (IQR), *months*	4.3 (2.7 to 7.8)	2.6 (2.1 to 3.4)	6.0 (4.4 to 8.9)	N/A	N/A
Ambulation status					
Walking with post-operative shoe, *n (%)*	21 (77.8)	10 (83.3)	11 (73.3)	0.55 (0.08 to 3.68)	0.662
Wheelchair bound, *n (%)*	2 (7.4)	1 (8.3)	1 (6.7)	0.79 (0.04 to 14.03)	> 0.99
Crutches, *n (%)*	3 (11.1)	1 (8.3)	2 (13.3)	1.69 (0.14 to 21.27)	> 0.99
Scooter, *n (%)*	1 (3.7)	0 (0)	1 (6.7)	0.93 (0.82 to 1.07)	> 0.99
Total number of complications/adverse events	21	7	14	N/A	N/A
Complications/adverse events, *n (%)*	16 (59.3)	6 (50.0)	10 (66.7)	2.00 (0.42 to 9.52)	0.452
Ulceration, *n (%)*	2 (7.4)	0 (0)	2 (13.3)	0.87 (0.71 to 1.06)	0.487
Amputation, *n (%)*	0 (0)	0 (0)	0 (0)	N/A	N/A
Infection, *n (%)*	0 (0)	0 (0)	0 (0)	N/A	N/A
Deep vein thrombosis, *n (%)*	0 (0)	0 (0)	0 (0)	N/A	N/A
Falls, *n (%)*	0 (0)	0 (0)	0 (0)	N/A	N/A
Asymmetry pain, *n (%)*	6 (22.2)	2 (16.7)	4 (26.7)	1.82 (0.27 to 12.17)	0.662
Rubbing/irritation, *n (%)*	11 (40.7)	5 (41.7)	6 (40.0)	0.93 (0.20 to 4.37)	> 0.99
Self-inflicted, *n (%)*	0 (0)	0 (0)	0 (0)	N/A	N/A
Other, *n (%)*^a^	2 (7.4)	0 (0)	2 (13.3)	0.87 (0.71 to 1.06)	0.487
None, *n (%)*	11 (40.7)	6 (50.0)	5 (33.3)	1.00 (0.20 to 5.00)	> 0.99
Treatment after TCC					
Specialised footwear and custom foot orthoses, *n (%)*	13 (48.1)	4 (33.3)	9 (60.0)	3.00 (0.62 to 14.62)	0.168
Life-long CROW, *n (%)*	7 (25.9)	5 (41.7)	2 (13.3)	0.22 (0.03 to 1.41)	0.185
Reconstructive/bone surgery, *n (%)*	4 (14.8)	1 (8.3)	3 (20.0)	2.75 (0.25 to 30.51)	0.605
Soft tissue surgery, *n (%)*	2 (7.4)	1 (8.3)	1 (6.7)	0.79 (0.04 to 14.03)	> 0.99
CAM walker, *n (%)*	1 (3.7)	1 (8.3)	0 (0)	1.09 (0.92 to 1.29)	0.444
Recurrent Charcot foot, *n (%)*	0 (0)	0 (0)	0 (0)	N/A	N/A
Contralateral Charcot foot, *n (%)*	3 (11.1)	1 (8.3)	2 (13.3)	1.69 (0.14 to 21.27)	> 0.99
Charcot pattern					
Tarsometatarsal joints, *n (%)*	1 (33.3)	0 (0)	1 (50.0)	N/A	N/A
NC, TN, CC joints, *n (%)*	1 (33.3)	0 (0)	1 (50.0)	N/A	N/A
Ankle and subtalar joints, *n (%)*	0 (0)	0 (0)	0 (0)	N/A	N/A
Combination, *n (%)*^b^	1 (33.3)	1 (100.0)	0 (0)	N/A	N/A

Data are n (%) or OR (95% CI), unless otherwise specified. Percentages may not add up to 100%, as they are rounded to the nearest percent

*CAM*, controlled ankle motion; CC, calcaneocuboid; *CI*, confidence interval; *CROW*, Charcot restraint orthotic walker; *IQR*, interquartile range; *N/A*, not applicable; *NC*, naviculocuneiform; *OR*, odds ratio; *SD*, standard deviation; *TCC*, total contact casting; *TN*, talonavicular

^*^Significant difference between ‘< 4 months’ and ‘> 4 months’ median TCC duration groups, p < 0.05

^a^One participant (3.7%) developed dermatitis. One participant (3.7%) experienced a plaster saw cut on removal of TCC

^b^One participant (3.7%) had a combination Charcot pattern involving the tarsometatarsal joints and the NC, TN and CC joints

On comparison of participants with more or less than 4 months duration of TCC treatment, only osteoarthritis was significantly associated with a longer TCC duration (OR, 6.00; 95% CI, 1.11 to 32.55; *p* = 0.031). All other patient and clinical factors were non-significant (Tables 1 to 4).

From acute Charcot diagnosis to resolution, the greatest reduction in dermal temperature differential was seen at the medial aspect of the navicular (− 1.7 °C), followed closely by the dorsal aspect of the base of the 3rd metatarsal, the medial aspect of the base of the 1st metatarsal, and the medial malleolus (− 1.5 °C) (Table 5).

**Table 5:** Dermal temperatures

Site	Anatomical location	Average temperature differential at acute Charcot diagnosis (°C)^a^	Average temperature differential at acute Charcot resolution (°C)^a^	Diagnosis minus resolution for average temperature differential (°C)^b^
1	Plantar 1st metatarsal head	2.5 (2.2)	1.3 (1.6)	1.2
2	Plantar 3rd metatarsal head	2.8 (2.0)	1.4 (2.2)	1.4
3	Plantar 5th metatarsal head	2.6 (2.2)	1.2 (1.8)	1.4
4	Plantar aspect of the base of the 5th metatarsal (styloid process)	2.4 (1.3)	1.1 (1.3)	1.3
5	Dorsal aspect of the base of the 3rd metatarsal	2.9 (2.2)	1.4 (1.5)	1.5
6	Medial aspect of the base of the 1st metatarsal	3.0 (2.1)	1.5 (1.8)	1.5
7	Medial aspect of the navicular	3.0 (1.7)	1.3 (1.3)	1.7
8	Plantar medial tubercle of the calcaneus	1.6 (1.4)	0.7 (1.1)	0.9
9	Medial malleolus	2.6 (1.7)	1.1 (1.3)	1.5
10	Lateral malleolus	2.9 (1.5)	1.7 (1.3)	1.2

Data are mean (SD), unless otherwise specified

*SD*, standard deviation

^a^Average temperature differential between the Charcot foot and the contralateral foot at specific anatomical sites

^b^Average temperature differential at diagnosis minus the average temperature differential at resolution (i.e. average temperature reduction from acute Charcot diagnosis to resolution)

## Discussion

This is one of the first studies to investigate duration of TCC treatment for resolution of acute Charcot foot in Australian subjects. Overall, this study found that the median duration of TCC treatment was 4.3 (IQR, 2.7 to 7.8) months. This finding is observed to be shorter than reports from studies in the UK (median, 9 to 12 months) [2, 17, 18], but is mostly comparable to studies conducted in the US (mean, 3 to 5 months) [10, 11, 19, 20], Europe (mean, 3 to 9 months) [21–27], and other Asia Pacific countries, including Thailand (median, 5 months) [30] and New Zealand (mean, 5 months) [29]. Interestingly, this finding was lower than an earlier Australian study conducted in Perth, where the average TCC duration was approximately 10 months [31]. This global (and local) variation may be explained by differing participant characteristics, Charcot characteristics (e.g. pattern and stage), techniques and protocols for monitoring Charcot progression, definition of Charcot resolution, the type of offloading used and their respective protocols (e.g. initial treatment with TCC and then transitioning to removable walker) [10, 25, 26], reduced access to services, staff capacity and experience in applying the TCCs, and study design [2, 20, 21, 43–46].

That being said, the available data on TCC duration are largely derived from retrospective and observational studies with small sample sizes, therefore, there is limited ability to determine temporal relationships with the reported clinical outcomes. The two largest studies in the literature includes a web-based observational study [2] with 288 acute Charcot cases from 76 centres in the UK and Ireland (although data on resolution were only available in 219 participants). The median duration of TCC treatment was 9 months [2]. Another study [28] that conducted a retrospective analysis on 164 participants with acute Charcot found the average TCC treatment time was 6 months [28]. Given that these two studies have the largest cohorts, and are likely to be the most clinically relevant, these figures may perhaps act as a benchmark for TCC duration more broadly.

There were five additional important findings from our study. First, those with osteoarthritis were 6-fold more likely to have a TCC duration of more than 4 months for acute Charcot foot resolution, compared to those without osteoarthritis (OR, 6.00; 95% CI, 1.11 to 32.55; *p* = 0.031). While there is some evidence to suggest a relationship between rheumatoid arthritis and Charcot neuroarthropathy [47], to the authors’ knowledge, no publications have reported Charcot neuroarthropathy in association with osteoarthritis. Our finding that osteoarthritis may be associated with longer TCC duration (i.e. longer time to Charcot resolution) may be explained by the biomechanical (e.g. foot structure and function) and/or biochemical factors (e.g. chemokines, cytokines, growth factors) associated with osteoarthritis [48], which may be further exacerbated by the Charcot process. This finding, however, should be interpreted with caution due to the wide confidence interval. There were no other significant patient or clinical factors that affected TCC treatment time (i.e. when comparing those < 4 or > 4 months TCC duration), which is consistent with a previous study [15]. Due to the small sample size (*n* = 27) and retrospective nature of this study, high-quality prospective studies with large sample sizes are needed to confirm these findings.

Second, our findings relating to Charcot foot characteristics, including Charcot pattern and history of misdiagnosis, are similar to reports from previous studies [21, 31]. In the current study, Charcot foot most commonly affected the tarsometatarsal joints (44.4%) or midfoot (40.7%). Overall, 85.2% were triggered by an ulcer or traumatic injury and were present for a median of 2.0 (IQR, 1.0 to 6.0) months prior to attending the HRFS. Charcot misdiagnosis occurred in 63.0% of participants prior to attending the HRFS, most commonly confused with cellulitis (47.1%). Despite increased awareness, the acknowledged importance of Charcot-related patient education [49], and the publication of an evidence-based pathway [3], disparity still remains for early diagnosis [31]. Our findings support close monitoring for signs of Charcot foot in those with ulceration or reported traumatic injury to the foot. Furthermore, in those presenting with a warm, erythematous and/or oedematous foot, Charcot diagnosis should be considered, and the foot treated as such, until proven otherwise [8]. This is particularly important for high-risk patients with long-standing diabetes and neuropathy [3, 21].

Third, of the 421 TCCs applied, there were 21 complications in total. This equates to an overall complication rate of 5% per cast, which is consistent with a previous study [50]. Overall, 59.3% of participants experienced a complication or adverse event, the majority being minor and reversible. The most common complications were skin rubbing/irritation (61.1%) and asymmetry pain (33.3%). Previous studies have shown that the duration of non-removable (e.g. TCC) versus removable offloading devices (e.g. Aircast, CAM walker) for Charcot resolution is significantly less [2, 31]. Given that the use of non-removable offloading can shorten the median time to resolution by approximately 3 months [2], this option may appeal to patients with Charcot foot due to the known physical, mental and social consequences of prolonged offloading (e.g. muscle atrophy, reduced activity levels and fitness, weight gain, poor glycaemic control, risk of falls, loss of work or income, offloading-related stigma, reduced health-related quality of life, inability to participate in certain family activities) [1, 5]. Our findings and those of a previous study [50] support that TCC is a relatively safe modality for offloading and immobilising neuropathic feet, despite some expected minor and reversible complications [50]. However, it is still essential when considering TCC treatment that the risks and benefits are carefully considered, and that patients are fully informed of these risks prior to their first TCC application.

Fourth, a large proportion of participants experienced good clinical outcomes post TCC treatment. Almost half were able to return to specialised or off-the-shelf footwear and custom foot orthoses (48.1%), while the other half with more severe cases of Charcot foot chose to wear a life-long CROW (25.9%) or had soft tissue or bone reconstructive surgery (22.2%). Fortunately, there were no recurrent Charcot feet recorded, which is consistent with other studies [15, 26]. Contralateral Charcot foot occurred in 3 (11.1%) participants. Therefore, to reduce the risk of bilateral Charcot, it is essential to ensure contralateral footwear is appropriate and an offloading foot orthotic is fitted [3]. As this study only collected data over a three-year period, longitudinal, prospective studies are needed to evaluate these clinical outcomes further.

Fifth, from acute Charcot diagnosis to resolution, the greatest reduction in temperature differential (i.e. when performing dermal temperature measurements at 10 commonly used anatomical sites) was seen at the medial aspect of the navicular (− 1.7 °C), followed closely by the dorsal aspect of the base of the 3rd metatarsal, the medial aspect of the base of the 1st metatarsal, and the medial malleolus (− 1.5 °C). This reduction in temperature differential is supported by a previous study [14]. Considering that the highest dermal temperatures often correlate with the joints affected by Charcot [3, 51, 52], this may explain why a greater temperature reduction was seen at these anatomical sites (i.e. as the majority of Charcot feet in this study affected the tarsometatarsal and midfoot joints). This finding provides valuable information regarding the expected reduction in temperature differential for each specific anatomical testing site at Charcot resolution, which may assist clinicians in monitoring Charcot progression.

This study has the limitations of a retrospective design and small sample size. To address the potential for selection bias, all participants that met the strict study eligibility criteria within the available sample were included. Therefore, the risk of selection bias was negligible or essentially non-existent. Due to the small sample size (*n* = 27), there is an increased likelihood of the study being underpowered, which may have increased the risk for type II statistical errors. For example, the ability to detect differences between the TCC groups (i.e. < 4 months and > 4 months duration). However, as all eligible participants were exhausted from the available sample, the maximum sample size was reached. Therefore, it was not possible to increase the sample size of the current study. Another limitation relates to having no data or measurements pertaining to the degree of adherence to the TCC treatment. However, as this was a non-removable device, poor adherence to treatment was unlikely. Regarding external validity, our findings are mostly generalisable to patients presenting with modified Eichenholtz stage 1 Charcot foot and those with diabetes (88.9% of the cohort). In addition, as this study only included participants from a single metropolitan health service, this may affect the applicability of the findings to the broader Australian context. Finally, in cases of unexpected removal (e.g. patient request, work/travel requirements), the time spent out of the TCC was included in the participant’s overall TCC treatment time. As a result, this may have slightly increased the study’s calculated median TCC duration. However, to ensure this study remained pragmatic and clinically relevant, the authors’ decision to include time spent out of the TCC was relevant.

Despite these limitations, this is one of the first studies to investigate duration of TCC treatment for resolution of acute Charcot foot in Australian subjects. This study had a rigorous inclusion and exclusion criteria, sound HRFS protocols using objective measurements (e.g. dermal temperatures and medical imaging findings) for monitoring the progression and resolution of acute Charcot, and expert TCC plaster technicians who used the same application technique for each cast (Fig. 1).

Large, high-quality, prospective studies are required to confirm the findings of this study and those reported in previous literature [2, 10, 11, 17–31]. Future research may be directed towards investigating TCC duration for:

(i) removable (e.g. CROW, CAM walker) versus non-removable (e.g. TCC, iTCC) offloading devices for Charcot treatment, including an evaluation of treatment adherence (e.g. installing an accelerometer into a removable offloading device, as per a previous study [53]);
(ii) non diabetes-related Charcot neuroarthropathy;
(iii) patients with/without diabetes or with varying diabetes duration and/or;
(iv) different stages of Charcot foot.

There is also a need for longitudinal studies to evaluate adverse clinical outcomes, such as Charcot recurrence rates, development of contralateral Charcot, and rates of Charcot-related foot ulcers, infections and amputations.

Importantly, this study provides insight into the duration of TCC treatment for resolution of acute Charcot foot in Australian subjects, with comparisons made to the global body of evidence. Patient characteristics, Charcot foot presentations, TCC complications, factors affecting TCC duration, and post-TCC clinical outcomes have also been explored. The findings may provide recommendations and assist clinicians in relaying evidenced-based education for patients newly diagnosed with Charcot foot. Further, the findings may also assist Australian metropolitan settings (i.e. that follow a similar treatment protocol to the one described in this study) by developing HRFS patient pathways and expected resource requirements, clinical decision making for offloading treatment plans, managing patient expectations and goals, developing risk reduction plans, and improving overall adherence to TCC treatment for acute Charcot neuroarthropathy cases in HRFS throughout Australia. However, it is important to consider these findings and possible applications in the context of the study’s limitations, the variation of TCC treatment times between the current study and an existing Australian study [31], and the broad TCC duration found in this study (IQR, 2.7 to 7.8 months).

## Conclusions

This is one of the first studies to investigate the duration of TCC treatment for resolution of acute Charcot foot in Australian subjects. The median time to resolution was 4 months, which is shorter or comparable to data reported in the UK, US, Europe, and other Asia Pacific countries. Osteoarthritis was significantly associated with a longer TCC duration. The findings from this study may assist clinicians in providing patient education, managing expectations and improving adherence to TCC treatment for acute Charcot neuroarthropathy cases in Australia. High-quality, prospective, longitudinal, multi-centre studies are now needed to confirm the findings of this study and to provide a broader application to the Australian context.

## Supplementary Information

**Additional file 1.** Adobe professional. Screening tool and data collection form. Screening tool used to identify eligible participants and data collection form used to collate the participant data.

## Data Availability

All data generated or analysed during this study are included in this published article and its supplementary information files.

## Abbreviations

CAM: Controlled ankle motion
CI: Confidence interval
CROW: Charcot restraint orthotic walker
HRFS: High-risk foot service
IQR: Interquartile range
iTCC: Instant total contact cast
OR: Odds ratio
SD: Standard deviation
TCC: Total contact casting
UK: United Kingdom
US: United States
