# Supplementary material for: Duration of total contact casting for resolution of acute Charcot foot: a retrospective cohort study
Source: J Foot Ankle Res. 2021 Jun 15;14:44. doi: 10.1186/s13047-021-00477-5 (PMC8204579; doi:10.1186/s13047-021-00477-5)
Supplement: Supplementary file 1 — Additional file 1. Adobe professional. Screening tool and data collection form. Screening tool used to identify eligible participants and data collection form used to collate the participant data. [file 13047_2021_477_MOESM1_ESM.pdf]

# SCREENING TOOL / DATA COLLECTION FORM

## SCREENING FOR ELIGIBILITY

| | | | | | |
|---|---|---|---|---|---|
| **Charcot episode of care between 4 January 2012 – 4 January 2015:** | Yes | ☐ | No | ☐ | *If NO, exclude* |
| **Acute Charcot foot:** **(i.e. Modified Eichenholtz stage 0 or 1)** | Yes | ☐ | No | ☐ | *If NO, exclude* |
| **≥18 years of age:** | Yes | ☐ | No | ☐ | *If NO, exclude* |
| **TCC treatment for ulcer or fracture:** **(i.e. not Charcot-related)** | Yes | ☐ | No | ☐ | *If YES, exclude* |
| **Concurrent rheumatic condition:** **(e.g. rheumatoid arthritis)** | Yes | ☐ | No | ☐ | *If YES, exclude* |
| **Concurrent inflammatory condition:** **(e.g. systemic lupus erythematosus)** | Yes | ☐ | No | ☐ | *If YES, exclude* |
| **Current foot infection:** **(e.g. cellulitis, erysipelas, osteomyelitis)** | Yes | ☐ | No | ☐ | *If YES, exclude* |
| **Transfer or withdrawal from HRFS:** **(i.e. between 4 January 2012 – 4 January 2015)** | Yes | ☐ | No | ☐ | *If YES, exclude* |

*** If participant satisfies all eligibility criteria, continue with assessment ***

# DATA COLLECTION

## 1. PARTICIPANT CHARACTERISTICS

**Date of assessment:** _______

**Date of birth:** / / **Sex:** Male ☐ *1* Female ☐ *0*

**Known ETOH abuse:** Yes ☐ *1* No ☐ *0*

**Smoking history:** Past ☐ *1* Current ☐ *2* Never ☐ *3*

## 2. COMORBIDITIES

**Diabetes mellitus:** Yes ☐ *1* No ☐ *0*

Type of diabetes: Type 1 ☐ *1* Type 2 ☐ *2*

Date of diagnosis: ___________ Duration of diabetes (years): ___________

Latest HbA1c result: ___________ (%)

**Dyslipidaemia:** Yes ☐ *1* No ☐ *0*

**Hypertension:** Yes ☐ *1* No ☐ *0*

**Ischaemic heart disease:** Yes ☐ *1* No ☐ *0*

**Congestive cardiac failure:** Yes ☐ *1* No ☐ *0*

**Cerebrovascular disease:** Yes ☐ *1* No ☐ *0*

**Chronic kidney disease:** Yes ☐ *1* No ☐ *0*

**Osteoarthritis:** Yes ☐ *1* No ☐ *0*

**Previous foot ulceration:** Yes ☐ *1* No ☐ *0*

**Previous foot infection:** Yes ☐ *1* No ☐ *0*

**Previous amputation:** Yes ☐ *1* No ☐ *0*

## 3. NEUROVASCULAR FOOT ASSESSMENTS

### 3.1 Neurological assessment

| Protective sensation (Semmes-Weinstein 5.07/10 g monofilament) | | | |
|---|---|---|---|
| | **Plantar Hallux** | **Plantar 3rd MTPJ** | **Plantar 5th MTPJ** |
| Left foot | | | |
| Right foot | | | |

*MTPJ*, Metatarsophalangeal joint.

✓ = present, × = absent (score /3 both feet)

*Note.* Inability to detect the monofilament at ≥1 site(s) will indicate peripheral neuropathy.

**Peripheral neuropathy:** Yes ☐ *1* No ☐ *0*

### 3.2 Arterial assessment

| Pedal pulses | | |
|---|---|---|
| | **Dorsalis pedis** | **Posterior tibial** |
| Left foot | | |
| Right foot | | |

✓ = present, × = absent (score /2 both feet)

*Note.* Absence of ≥2 pedal pulses on both feet indicates peripheral arterial disease.

| Ankle-brachial pressure index (ABPI) and toe-brachial pressure index (TPBI) | | |
|---|---|---|
| | **Left** | **Right** |
| Toe systolic pressure (mmHg) | | |
| Ankle systolic pressure (mmHg) | | |
| Brachial systolic pressure (mmHg) | | |
| ABPI value | | |
| TBPI value | | |

*ABPI*, Ankle brachial index; *TBPI*, Toe brachial index.

*Note.* ABPI ≤0.9 and/or TBPI ≤0.6 indicates peripheral arterial disease. ABPI >1.3 or non-compressible arteries (i.e. >240 mm Hg) indicates medial arterial calcification.

**Peripheral arterial disease:** Yes ☐ *1* No ☐ *0*

## 4. CHARCOT FOOT HISTORY

**Left foot** ☐ 1 **Right foot** ☐ 2 **Bilateral** ☐ 3

**Stage of Charcot:** Eichenholtz stage 0 ☐ 1 Eichenholtz stage 1 ☐ 2

**Date of diagnosis:** ___________

**Duration of Charcot foot (months):** ___________

**Charcot trigger:**

☐ 1 Ulceration
☐ 2 Injury/trauma
☐ 3 Amputation
☐ 4 Lymphoedema
☐ 5 Unknown

**Misdiagnosis of Charcot foot:** Yes ☐ 1 No ☐ 0
**(i.e. prior to attending HRFC)**

**Charcot foot pattern:**

☐ 1 Forefoot joints
☐ 2 Tarsometatarsal joints
☐ 3 Naviculocuneiform, talonavicular and calcaneocuboid joints
☐ 4 Ankle and subtalar joints
☐ 5 Calcaneus
☐ 6 Combination___________ (describe)

**Diagnostic imaging:**
**X-ray:** Yes ☐ 1 No ☐ 0

Initial date of imaging: ___________ Latest date of imaging: ___________

**Bone scan:** Yes ☐ 1 No ☐ 0

Initial date of imaging: ___________ Latest date of imaging: ___________

**MRI:** Yes ☐ 1 No ☐ 0

Initial date of imaging: ___________ Latest date of imaging: ___________

## 5. TOTAL CONTACT CASTING TREATMENT

**TCC start date:** ___________ **TCC cessation date:** ___________

**Duration of TCC treatment (months):** ___________

**Total number of TCC applications:** ___________

**Ambulation status (while in TCC):**

☐ 1 Walking with post-op shoe
☐ 2 Wheelchair bound
☐ 3 Crutches
☐ 4 Scooter
☐ 5 Other

**TCC complications/adverse events:** Yes ☐ 1 No ☐ 0

Types of complications/adverse events:

☐ 1 Ulceration
☐ 2 Amputation
☐ 3 Infection
☐ 4 Deep vein thrombosis
☐ 5 Fall(s)
☐ 6 Asymmetry pain (e.g. hip, back, knee, ankle pain)
☐ 7 Rubbing/irritation
☐ 8 Self-inflicted (e.g. placing objects within TCC, self-modification of TCC)
☐ 9 Other

Total number of TCC complications/adverse events: ___________

**Treatment after TCC:**

☐ 1 Specialised footwear and custom foot orthoses
☐ 2 Charcot Restraint Orthotic Walker (CROW)
☐ 3 Reconstructive/bone surgery
☐ 4 Soft-tissue surgery
☐ 5 CAM Walker

**Recurrent Charcot foot:** Yes ☐ 1 No ☐ 0

Charcot foot pattern:

☐ 1 Forefoot joints
☐ 2 Tarsometatarsal joints
☐ 3 Naviculocuneiform, talonavicular and calcaneocuboid joints
☐ 4 Ankle and subtalar joints
☐ 5 Calcaneus
☐ 6 Combination___________ (describe)

**Contralateral Charcot foot:** Yes ☐ 1 No ☐ 0

Charcot foot pattern:

☐ 1 Forefoot joints
☐ 2 Tarsometatarsal joints
☐ 3 Naviculocuneiform, talonavicular and calcaneocuboid joints

☐4 Ankle and subtalar joints
☐5 Calcaneus
☐6 Combination___________ (describe)

## 6. DERMAL TEMPERATURES

| Site | Anatomical location | Temperature differential (°C): Charcot diagnosis | Temperature differential (°C): Charcot resolution |
|---|---|---|---|
| **1** | Plantar 1st metatarsal head | | |
| **2** | Plantar 3rd metatarsal head | | |
| **3** | Plantar 5th metatarsal head | | |
| **4** | Plantar aspect of the base of the 5th metatarsal (styloid process) | | |
| **5** | Dorsal aspect of the base of the 3rd metatarsal | | |
| **6** | Medial aspect of the base of the 1st metatarsal | | |
| **7** | Medial aspect of the navicular | | |
| **8** | Plantar medial tubercle of the calcaneus | | |
| **9** | Medial malleolus | | |
| **10** | Lateral malleolus | | |
